# IL-22-producing Th22 cells play a protective role in CVB3-induced chronic myocarditis and dilated cardiomyopathy by inhibiting myocardial fibrosis

**DOI:** 10.1186/s12985-014-0230-z

**Published:** 2014-12-30

**Authors:** Yujie Guo, Weifeng Wu, Zhihong Cen, Xiaomo Li, Qing Kong, Qiuxi Zhou

**Affiliations:** Department of Cardiology, First Affiliated Hospital of Guangxi Medical University, Guangxi Cardiovascular Institute, Nanning, 530021 China

**Keywords:** Th22 cells, IL-22, Myocardial fibrosis, Chronic myocarditis, Dilated cardiomyopathy

## Abstract

**Background:**

A new subset of T helper (Th) cells, named IL-22-producing Th22 cells, was identified recently. Th22 cells have been implicated in immunity and inflammation. However, the role of these cells in the progression from acute viral myocarditis (AVMC) to dilated cardiomyopathy (DCM) and myocardial fibrosis remains unknown.

**Methods:**

BALB/c mice were repeatedly i.p. infected with Coxsackie virus B3 (CVB3) to establish models of AVMC, chronic myocarditis and DCM. On week 2, 12 and 24 post initial injection, the percentage of splenic Th22 cells, the levels of plasma IL-22, cardiac IL-22 receptor (IL-22R) expression, and indicators of myocardial fibrosis were measured. Further, mice with AVMC and chronic myocarditis were treated with an anti-IL-22 neutralizing antibody (Ab). The collagen volume fraction (CVF), the percentage of splenic Th22 cells, plasma IL-22 levels, cardiac IL-22R expression and indicators of myocardial fibrosis were then monitored.

**Results:**

Compared to control mice at the same time points, AVMC, chronic myocarditis and DCM mice have higher percentage of splenic Th22 cells, higher plasma IL-22 levels, increased cardiac IL-22R, as well as increased collagen typeI-A1 (COL1-A1), collagen type III-A1 (COL3-A1) and matrix metalloproteinase-9 (MMP9) expression. However, the expression of tissue inhibitor of metalloproteinase-1(TIMP-1) was decreased. Treatment of AVMC and chronic myocarditis mice with an anti-IL-22 Ab decreased the survival rate and exacerbated myocardial fibrosis. The percentage of splenic Th22 cells, plasma IL-22 levels and cardiac IL-22R expression also decreased in anti-IL-22 Ab treatment group as compared to IgG and PBS treated groups of AVMC and chronic myocarditis mice. Moreover, increased expression of COL1-A1, COL3-A1, MMP9 but decreased expression of TIMP-1 were observed in anti-IL-22 Ab mouse group.

**Conclusions:**

Th22 cells play an important role in the pathogenesis of CVB3-induced mouse chronic myocarditis and DCM. IL-22 is a myocardium-protective cytokine by inhibiting myocardial fibrosis. Therefore, Th 22 cells may be considered as potential therapeutic targets for DCM.

## Introduction

Viral myocarditis (VMC) is a common cardiac disease, characterized by myocardial inflammation due to virus infection. It was confirmed that the persistence of viral infection exists in some individuals with chronic myocarditis and dilated cardiomyopathy (DCM). Some patients with VMC may progress to chronic myocarditis and DCM, a terminal condition of heart failure and heart transplantation [1].

Emerging evidence has demonstrated that myocardial fibrosis is a major determinant in the development from VMC to DCM [2-4]. But the mechanism of myocardial fibrosis in disease procession has not been elucidated. It has been reported that T helper (Th) 1- and Th17-cell mediated autoimmune destruction may play an important role in myocardial fibrosis in which VMC progresses to DCM. However, Th1 and Th17 cell subsets may not fully explain the disease mechanism, because results from clinical trial and animal experiments concerning these T cell subsets were inconsistent [5-8].

Th22 cells are a subset of CD4^+^ effector T cells that primarily secrete IL-22. These cells do not express IL-17, IL-4, or IFN-γ [9,10]. IL-22 exerts its effect through IL-22R, which is a heterodimeric transmembrane receptor complex consisting of IL-22R1 and IL-10R2 [11]. Th22 cells play a key role in autoimmune tissue injury, including organ-specific autoimmunity [9]. Our previous studies have found that CVB3-induced AVMC mice have higher number of IL-22-producing Th22 cells and IL-22 shows critical anti-inflammatory and antiviral activity in disease development [12].

However, the role of Th22 cells and the mechanism of myocardial fibrosis in the course from AVMC to DCM are not clear. Therefore, our present study attempted to detect the percentage of Th22 cells, plasma IL-22 levels and cardiac IL-22R expression at stage of chronic myocarditis and DCM. We further explored the effect of neutralizing anti-IL-22 antibody (Ab) on myocardial fibrosis. Our study provided new insights into the role of Th22 cells in chronic myocarditis and DCM.

## Results

### Evaluation of the severity of AVMC, chronic myocarditis and DCM

**Figure 1:**
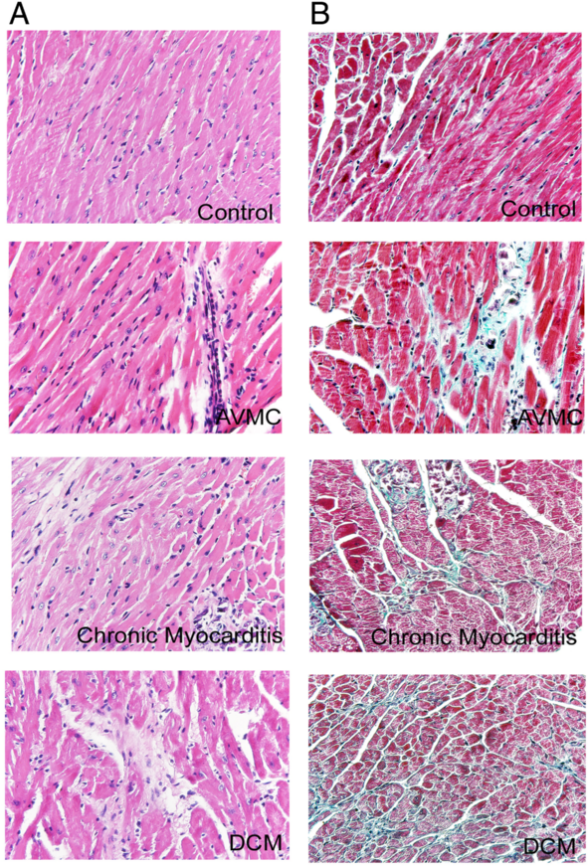
**The severity of AVMC, chronic myocarditis and DCM. A**. Representative myocardial histopathologic images in AVMC, chronic myocarditis and DCM (H&E, original magnification × 400). **B**. Representative myocardial fibrosis images in AVMC, chronic myocarditis and DCM (Masson’s Trichrome, original magnification × 400).

The characteristic signs in mice with AVMC, chronic myocarditis and DCM, including weakness, weight loss, irritability, back arching, coat ruffling, and lethargy were observed. After initial viral injection, 4 of 20 mice died in the AVMC group, 6 of 20 and 11 of 30 mice died in the chronic myocarditis and DCM groups. In contrast, no mice died in control groups. In sections of heart tissues from AVMC mice, larger numbers of inflammatory cells and necrosis with destruction of myocardial fibers were observed. At this stage, myocardial fibrosis was not visible. Inflammatory cells then decreased and myocardial collagen fibers increased with compensatory hypertrophy of myocardial cells. Necrotic myocardial cells gradually replaced by fibrous tissue at the stage of chronic myocarditis. In DCM mice, myocardial fibers were disorganized, with no inflammatory cell infiltration. Cardiomyocytes were seperated into several patchy areas by a numerous myocardial collagen fibers (Figure 1). The results indicate that the severity of myocardial fibrosis gradually increased with progression from AVMC to DCM.

### Exacerbation of myocardial fibrosis from AVMC to DCM

**Figure 2:**
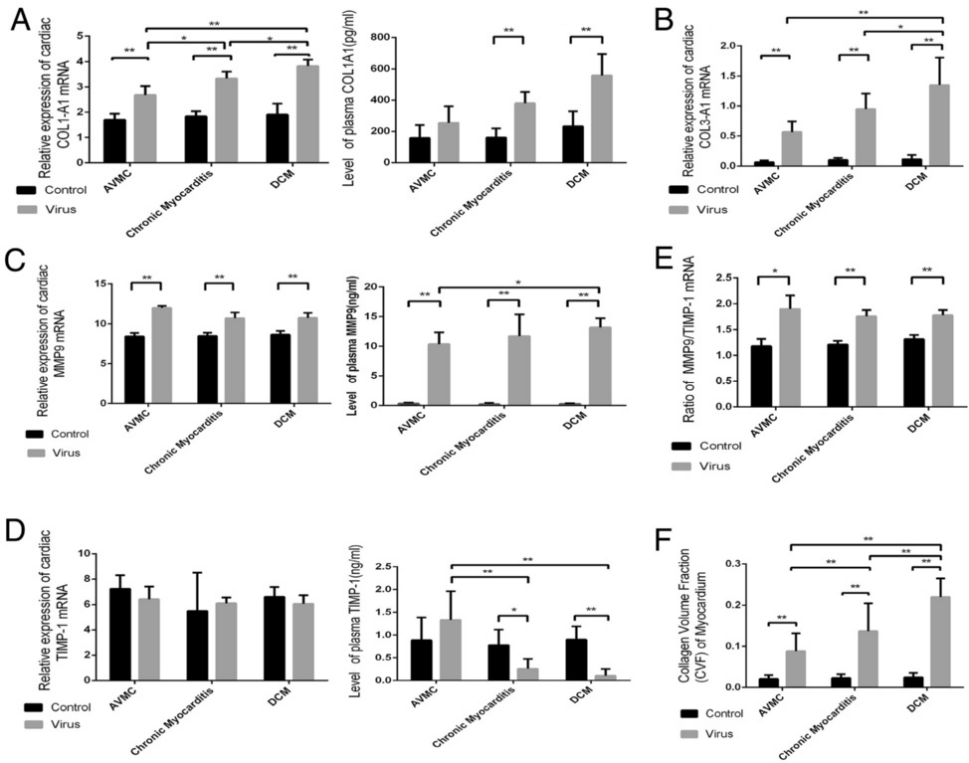
**Aggravated myocardial fibrosis from AVMC to DCM. A**. Cardiac expression of COL1-A1 was investigated by RT-PCR, and the level of circulating protein was measured by ELISA. **B**. Cardiac COL3-A1 expression in mice treated with virus. **C-D**. Cardiac MMP9 and TIMP-1 expression and circulating protein. **E**. The ratio of MMP9/TIMP-1 mRNA. **F**. CVF of mice receiving virus showing a progressive increase in the course from AVMC to DCM. *p < 0.05, **p < 0.01. Data are means ± SD.

The expression of collagen typeI-A1 (COL1-A1) gene in myocardium significantly elevated in all virus infected mice by comparison to control mice at the same time points (Figure 2A). In addition, the expression of COL1-A1 gene increased with the progression from AVMC to DCM. As shown in Figure 2A, compared to control mice, the levels of circulating COL1-A1 were significantly higher in mice with chronic myocarditis and DCM. Similarly, the expression of collagen type III-A1 (COL3-A1) gene in myocardium significantly elevated in mice treated with CVB3 (Figure 2B). Compared with control mice, higher levels of cardiac matrix metalloproteinase-9 (MMP9) gene and circulating MMP9 were detected in AVMC, chronic myocarditis and DCM mice (Figure 2C). We also observed alterations of cardiac tissue inhibitor of metalloproteinase-1(TIMP-1) gene and plasma TIMP-1. Data in Figure 2D confirmed that the levels of circulating TIMP-1 markedly decreased in mice with chronic myocarditis and DCM. The expression of TIMP-1 mRNA was not statistically different. Figure 2E shows that much higher ratio of MMP9/TIMP-1 was detected in AVMC, chronic myocarditis and DCM mice. Higher collagen volume fraction (CVF) was observed in mice receiving virus, revealing progressive increase in the course from AVMC to DCM (Figure 2F). With the progression from AVMC to DCM, the severity of myocardial fibrosis significantly increased accompanied by up-regulation of COL1-A1, COLA3-A1, MMP9 and ratio of MMP9/TIMP-1 but down-regulation of TIMP-1.

### Increased proportions of splenic Th22, plasma IL-22 levels and myocardial IL-22R expression in AVMC, chronic myocarditis and DCM mice

**Figure 3:**
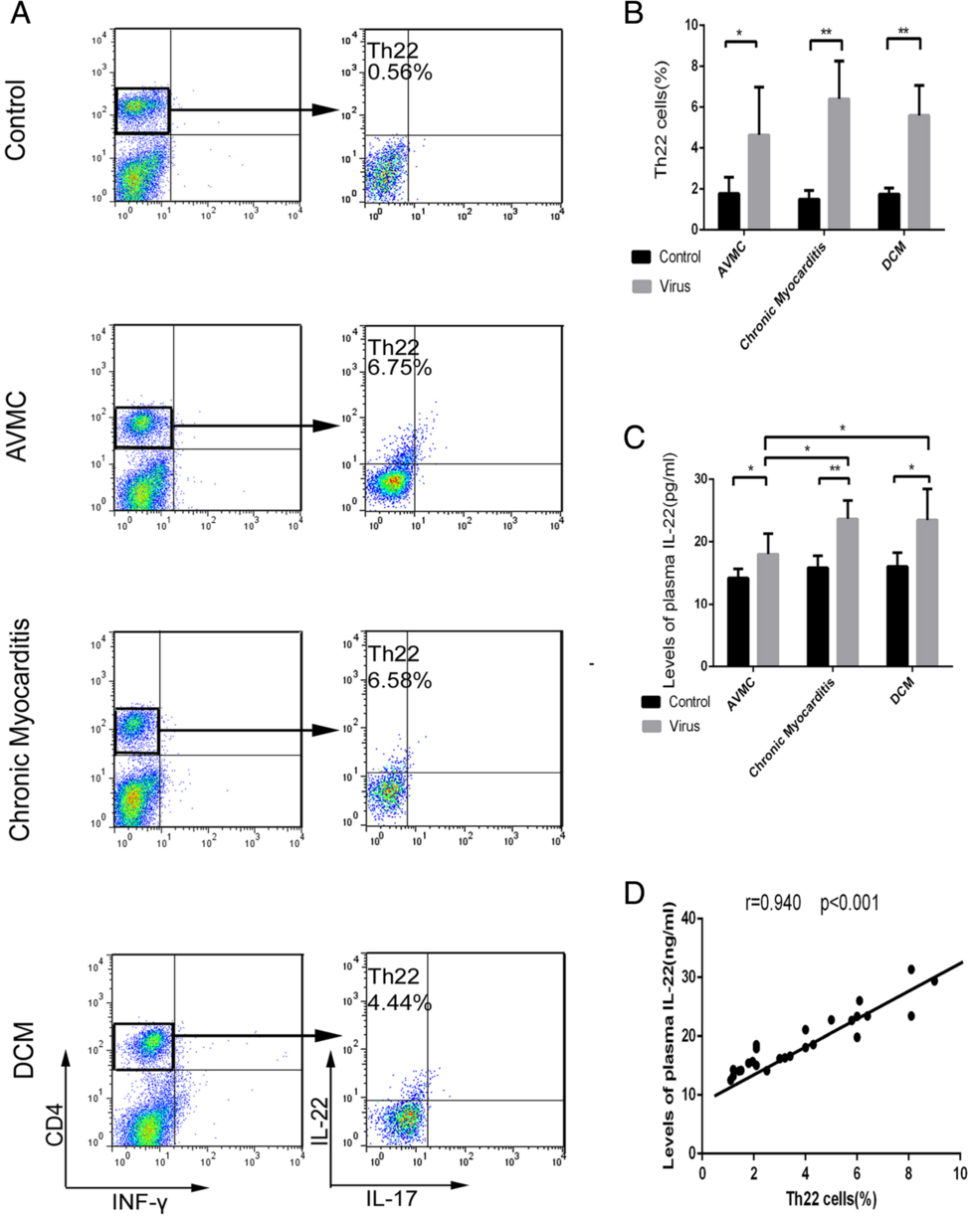
**Increased percentage of Th22 cells and circulating IL-22 in AVMC, chronic myocarditis and DCM. A**. Th22 cells identified based on expressions of IL-22 + IL-17-IFN-γ-CD4 +. **B**. Comparisons of Th22 cells in mice of AVMC, chronic myocarditis and DCM groups with control mice at the same time points. **C**. Up-regulation of circulating IL-22 detected in AVMC, chronic myocarditis and DCM mice. **D**. Correlation of the percentage of splenic Th22 cells with circulating IL-22.

**Figure 4:**
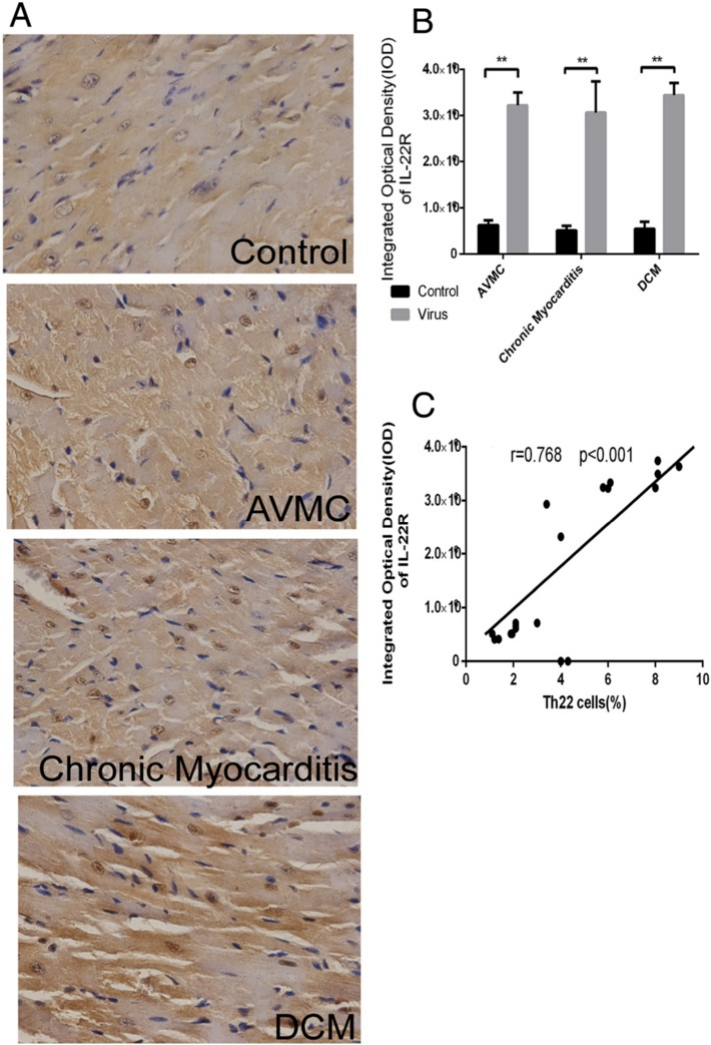
**Up-regulated cardiac IL-22R expression in AVMC, chronic myocarditis and DCM. A**. Representative IL-22R immunohistochemistry images in heart tissue (Dark brown granules, original magnification × 400). **B**. Morphometric quantitation of cardiac IL-22 protein. **C**. Correlation of the percentage of splenic Th22 cells with cardiac IL-22R. *p < 0.05, **p < 0.01. Data are the means ± SD.

Flow cytometry was used to analyze mononuclear cells from freshly isolated spleen of mice week 2, 12 and 24 post initial viral injection (Figure 3A). Figure 3B shows that the percentage of Th22 cells (IL-22 + IL-17-IFN-γ-CD4+) increased in mice with AVMC (4.64 ± 2.35%), chronic myocarditis (5.90 ± 1.74%) and DCM (5.60 ± 1.47%) relative to control mice (1.78 ± 0.79%, 1.50 ± 0.44%, 1.75 ± 0.30%, respectively). However, compared to chronic myocarditis mice, the percentage of splenic Th22 cells in DCM mice exhibited only a slight decrease. Th22 cell numbers among three mouse groups treated with virus were of no statistically significant difference. The levels of plasma IL-22 in mice with AVMC, chronic myocarditis and DCM markedly increased as compared to control mice (Figure 3C). Also, the expression of IL-22R protein was up-regulated in AVMC, chronic myocarditis and DCM mouse groups, compared to control mice (Figure 4A and B). Spearman rank correlation coefficients shown in Figures 3D and 4C demonstrated that the percentage of Th22 cells positively correlated with circulating IL-22 levels and cardiac IL-22R protein. Figures 3 and 4 show higher proportions of splenic Th22 cells, plasma IL-22 levels and myocardial IL-22R expression in AVMC, chronic myocarditis and DCM mice.

### Neutralization of IL-22 exacerbated the severity of AVMC and chronic myocarditis and the procession to DCM

**Figure 5:**
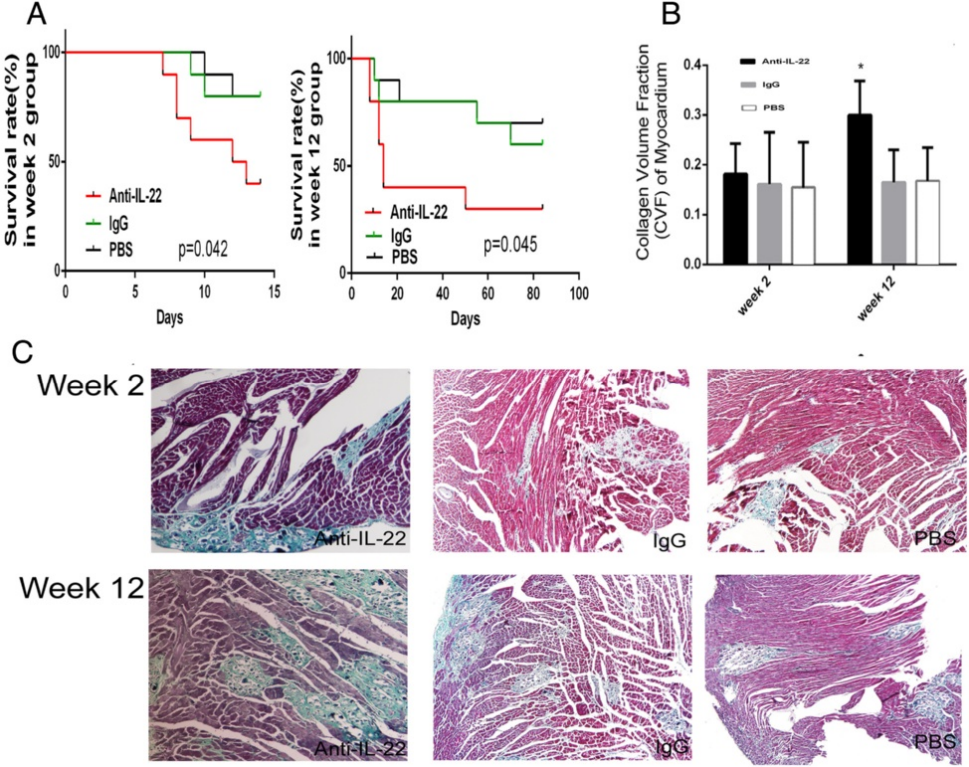
**Neutralization of IL-22 exacerbated the severity of AVMC and chronic myocarditis. A**. Survival rate of anti-IL-22 Ab treated mice at week 2 and 12 after neutralization of IL-22. **B**. CVF of mice in anti-IL-22 Ab subgroup at week 12. **C**. Representative myocardial fibrosis images in anti-IL-22 Ab, IgG and PBS treated mice at week 2 and 12 (Masson’s Trichrome, original magnification × 100).

Four out of 10 mice and 3 out of 10 mice survived at 2 and 12 weeks after viral infection in anti-IL-22 treatment subgroup. Eight out of 10 mice and 6 out of 10 mice survived at 2 and 12 weeks after initial infection in IgG treatment subgroup and 8 out of 10 mice and 7 out 10 mice in the PBS treated subgroup. Compared to IgG and PBS treatment subgroups, the survive rate of mice treated with anti-IL-22 Ab at week 2 and 12 was significant lower (Figure 5A). However, there was no difference in survival rate in IgG and PBS treatment subgroups. The CVF of myocardium in anti-IL-22 Ab treated mice exhibited a moderate increase compared to other mouse groups at week 2, but without statistical significance. Higher CVF was detected in mice receiving anti-IL-22 Ab at week 12 (Figure 5B). The increased level of CVF was similar to DCM mouse group (0.30 ± 0.07 vs. 0.24 ± 0.09, *p* > 0.05). The difference of CVF in IgG and PBS mouse subgroups was not significant. Figure 5C showed the severity of myocardial fibrosis in week 2 and 12 mouse groups. Anti-IL-22Ab treatment exacerbated the severity of AVMC and chronic myocarditis and accelerated the procession to DCM.

### Neutralization of IL-22 decreased the percentage of splenic Th22 cells, plasma IL-22 levels and cardiac IL-22R expression

**Figure 6:**
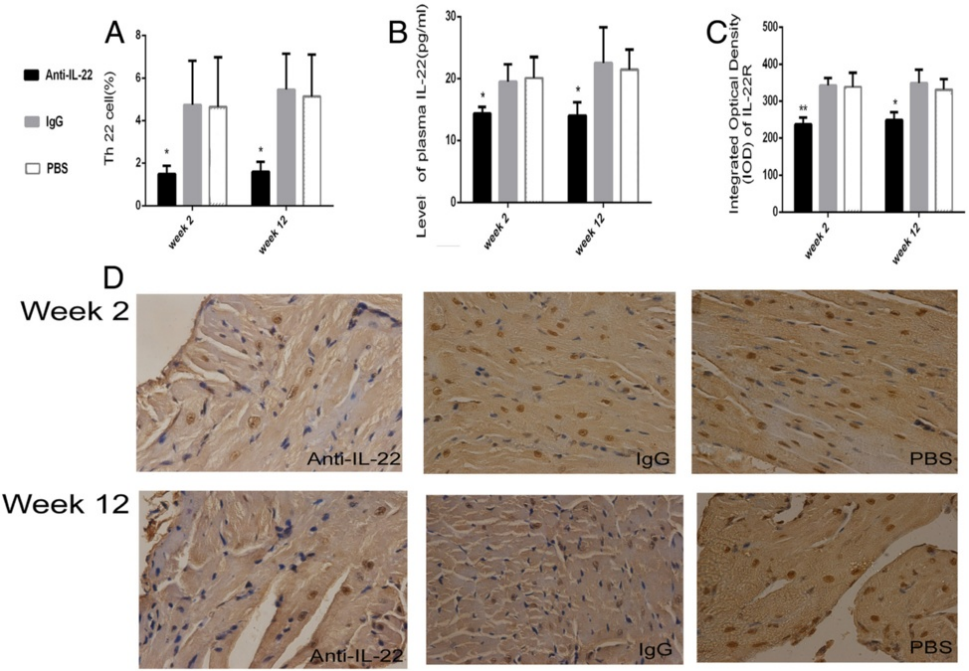
**Neutralization of IL-22 decreased the percentage of the Th22 cells, IL-22 and IL-22R. A**. Th22 cells in anti-IL-22 Ab, IgG and PBS treated mouse groups were investigated by flow cytometry at week 2 and 12. **B**. The levels of circulating IL-22 measured by ELISA. **C**. Morphometric quantitation of cardiac IL-22R protein expression detected by IHC. **D**. Representative IL-22R immunohistochemistry images in heart tissue in anti-IL-22 Ab, IgG control and PBS treated mouse groups (Dark brown granules, original magnification × 400).

The percentage of splenic Th22 cells was examined at week 2 and 12 (Figure 6A). Th22 cells in mice treated with anti-IL-22 Ab was fewer than IgG and PBS-treated subgroups (*p* < 0.05). As shown in Figure 6B, C and D, a marked reduction in circulating IL-22 and IL-22R expression in myocardium were observed in anti-IL-22 Ab treated mice. Th22 cells, IL-22 and IL-22R in IgG and PBS treated mouse subgroups remained at higher levels. There was no significant difference between IgG and PBS treated mice. Figure 6 showed that the percentage of splenic Th22 cells, circulating IL-22 and cardiac IL-22R expression decreased after neutralization of IL-22.

### Neutralization of IL-22 exacerbated myocardial fibrosis

**Figure 7:**
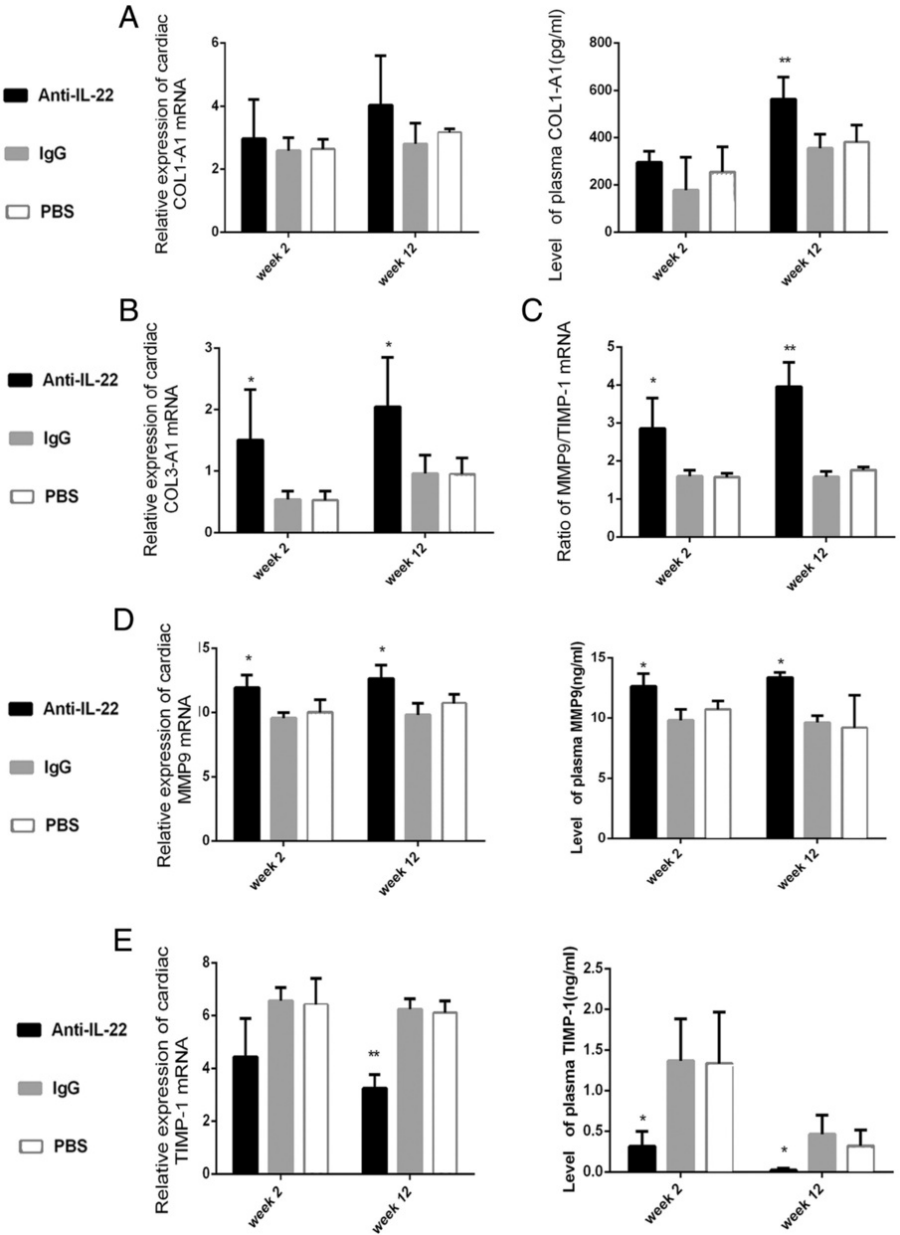
**Neutralization of IL-22 exacerbated myocardial fibrosis at stages of AVMC and chronic myocarditis. A**. Relative cardiac expression and plasma levels of COL1-A1 detected at week 2 and 12, measured by RT-PCR and ELISA. **B**. Up-regulation of cardiac expression of COL3-A1 in anti-IL-22 Ab treated mice. **C**. The ratio of MMP9/TIMP-1 mRNA. **D-E**. Higher MMP9 and lower TIMP-1 detected in anti-IL-22 Ab treated mice. *p < 0.05, **p < 0.01. Data are the means ± SD.

After neutralization of IL-22, the indicators of myocardial fibrosis were investigated at week 2 and 12. The levels of circulating COL1-A1 significantly elevated in anti-IL-22 Ab treated subgroup by comparison to IgG and PBS treated subgroups at week 12 (Figure 7A). As shown in Figure 7A, in anti-IL-22 Ab treated subgroup, the levels of circulating COL1-A1 was higher at week 2 and the expression of COL1-A1 gene in myocardium at week 2 and 12 was up-regulated, but without statistical significance compared to other mouse subgroups. Myocardium COL3-A1 mRNA was elevated in anti-IL-22 Ab treated mice at week 2 and 12 (Figure 7B). At week 12, the up-regulation of COL3-A1 gene in anti-IL-22 Ab-treated mice was similar to DCM mice. Figure 7C showed that, at week 2 and 12, much higher ratio of MMP9/TIMP-1 was observed in anti-IL-22 Ab treated mouse subgroup. Much higher levels of MMP9 transcripts in myocardium and circulating MMP9 were detected in anti-IL-22 Ab treated mouse subgroup at week 2 and 12 (Figure 7D, both *p* < 0.01). Data in Figure 7E confirmed decreased circulating TIMP-1 in mice receiving anti-IL-22 Ab at week 2 and 12, compared to IgG and PBS treated mice. The expression of TIMP-1 mRNA showed a little decrease in anti-IL-22 Ab treated mice at week 2 (*p* > 0.05). However, at week 12, marked increase was observed (*p* < 0.01). Data in this figure showed that statistically significant difference in the levels of COL1-A1, COL3-A1, MMP9, TIMP-1 and MMP9/TIMP-1 between IgG control and PBS subgroups was not been investigated on week 2 and week 12. Neutralization of IL-22 exacerbated myocardial fibrosis with down-regulation of the percentage of splenic Th22 cells, circulating IL-22 and myocardial IL-22R expression.

## Discussion

VMC is a common cardiac disease and some patients with VMC may progress to chronic myocarditis and DCM, with a terminal condition of heart failure requiring heart transplantation. The main characteristic of DCM is extensive myocardial fibrosis, due to cardiomyocyte death and accumulation of extracellular matrix (ECM) protein. The main collagens in ECM are type I (50–85%) and type III (10–45%) collagens [13]. We observed that the degree of myocardial fibrosis gradually increased with the progression from AVMC to DCM, accompanied by up-regulation of COL1-A1 and COLA3-A1. The role of MMP9 in infectious and autoimmune diseases was not clear, research with inconsistent reports [14-17]. TIMPs are inhibitors of MMPs, which disturb the pathological consequences through MMPs [18]. The MMP/TIMP-1 ratio is correlated to the heart injury. Our data showed increased MMP9 level, MMP9/TIMP-1 ratio and decreased TIMP-1 level in AVMC, chronic myocarditis and DCM mice. Up-regulation of MMP9, MMP9/TIMP-1 ratio and down-regulation of TIMP-1 were more significant after neutralizing IL-22, associated with myocardial fibrosis exacerbation. Therefore, MMP-9 may act as a damaging factor in the pathogenesis of DCM.

Growing evidence suggests that Th22 cells may play an important role in autoimmune disease such as systemic lupus erythematosus [19], oral and cutaneous Lichen Planus lesions [20], neuromyelitis optica, multiple sclerosis [21], psoriasis, psoriatic arthritis [22,23] and IgA nephropathy [24], as well as infectious disease including chronic HIV infection [25] and hepatitis [26,27]. Recently, Th22 cells have been implicated in endocrine and cardiovascular diseases, such as type 1 diabetes [28], type 2 diabetes and obesity [29], acute coronary syndrome [30], coxsackievirus B3 (CVB3)-induced acute viral myocarditis [12] and DCM [31].

Our previous study showed that Th22 cells and IL-22 may be a double-edged sword. Th22 cells and IL-22 have critical anti-inflammatory and antiviral effect in CVB3-induced mouse AVMC [12]. However, in IL-17A-deficient mice, IL-22 exacerbates CVB3-induced acute viral myocarditis [32]. Similar results were reported [33] concerning either protective and proinflammatory effects of Th22 cells. Our data showed that, compared with control mice at the same time points, the percentage of the pure Th22 cell, plasma IL-22 and cardiac protein expression of IL-22R were up-regulated in AVMC, chronic myocarditis and DCM groups. Then the myocardial fibrosis increased and survival rate decreased in mice after neutralizing IL-22, accompanied by a decline in Th22 cell number, reduced IL-22, diminished IL-22R expression and increased indicators of myocardial fibrosis such as COL1-A1, COL3-A1, MMP9. Moreover, the severity of myocardial fibrosis in chronic myocarditis after neutralizing IL-22 is similar to DCM. These results revealed that neutralization of IL-22 exacerbated the severity of AVMC and chronic myocarditis, aggravated myocardial fibrosis and accelerated the procession of DCM. Thus, Th22 cells and IL-22 play a protective role in DCM by inhibiting myocardial fibrosis and act as therapeutic targets.

However, there are several limitations in our study. First of all, the small number of mice, especially survival mice after neutralization of IL-22, may have a causal effect on outcome. In addition, the circulating IL-22 in vivo may not be completely antagonized using the injected dose of anti-IL-22 Ab. Other cells such as NKT, Th17 etc. can produce IL-22, those cells may interfere with the study of th22 cell function. As part of future efforts, it will be important to increase the number of mice and eliminate circulating IL-22 as much as possible, knockout mice may be used if necessary to confirm our results further.

## Conclusions

In summary, our study demonstrated that the up-regulations of IL-22-producing Th22 cells may play an important part in the pathogenesis of CVB3-induced mice chronic myocarditis and DCM by inhibiting myocardial fibrosis. IL-22 may serve as a myocardium-protective cytokine by means of decreasing COL1-A1, COL3-A1, MMP9 and increasing TIMP-1. In order to explore the therapeutic potential of Th22 cells in the progression from AVMC to DCM, future studies, which focus on regulating the downstream pathways of Th22 cells would be more promising.

## Methods

### Mice

Pathogen-free male BALB/c mice aged 4 week were purchased from the Guangdong Medical Laboratory Animal Centre, Foshan, China (Certificate No. SCXK (Yue) 2008–0002). All animals were kept in pathogen-free mouse room in Experimental Animal Center of the Guangxi Medical University, Nanning, China. All experiments were carried out in accordance with protocols approved by Guangxi Medical University Animal Ethics Committee.

### Virus

CVB3 (Nancy strain, from Institute of immunology of Guangxi Medical University) was maintained by passaging through Hep-2 cells. Virus titer was determined by plaque-forming unit (PFU) assay at 1 × 10^8^. CVB3 was diluted in phosphate-buffered saline (PBS) (Solarbio Science &Technology Co, Ltd, Beijing, China).

### Induction of AVMC, chronic myocarditis and DCM

BALB/c mice were infected by intraperitoneal injection (i.p) of 100 μl PBS containing approximately ~ 10^6^ PFUs of the virus for establishing AVMC. For chronic myocarditis and DMC, BALB/c mice were infected by the same dose of virus on day 0. Subsequently the virus was injected once every 4 weeks in increments of 10 μl in a total of three doses for establishing chronic myocarditis and six doses for establishing DCM.

One hundred BALB/c mice were randomly divided into four groups: 1) 20 in the AVMC group; 2) 20 in chronic myocarditis group; 3) 20 in DCM group; and 4) the rest mice as a PBS injection control group (n = 30). All surviving animals with AVMC and 10 control mice were sacrificed by the end of week 2 after infection. In chronic myocarditis and DCM, surviving animals and 10 control mice were sacrificed by the end of week 12 and 24, respectively. Heart and spleen were removed aseptically and blood was harvested to obtain plasma.

### Neutralization of IL-22

A total of 60 mice infected with CVB3 were randomly divided into two groups: 1) week 2 group, in which all surviving animals were sacrificed on day 14 after CVB3 infection, and 2) week 12 group, on day 84 after treatment with CVB3, all surviving mice were sacrificed. Each mouse group was separated into three subgroups: i.p. administration of anti-IL-22 Ab (50 μg per mouse every 4 weeks, at day 0, day 28 and day 56, n = 10, anti-IL-22Ab subgroup); normal IgG control (50 μg per mouse; n = 10, IgG control subgroup) and PBS (50 μg per mouse; n = 10, PBS subgroup). Anti-IL-22 Ab and normal IgG were from R&D Systems, Inc. Minneapolis. The survival rates of each mouse group were recorded and plasma, hearts and spleens of surviving mice were harvested on day 14 in week 2 group and day 84 in week 12 group.

### Histopathology

The left-ventricular tissues of the mouse heart were fixed in 10% formalin, then were embedded in paraffin. The tissues were incised into 5-μm sections along the length of the heart, and stained with H&E (hematoxylin and eosin) or Masson’s Trichrome. Histopathological changes were observed (Olympus BX53 Microscope, Tokyo, Japan).

### Flow cytometry

Spleens from mice were collected to prepare a single-cell suspension. The cells were resuspended in RPMI 1640 (Wisent, Nanjing, China) medium with 10% Superior Placental Bovine Serum (sijiqing, Hangzhou, China) and stimulated with phorbol myristate acetate (PMA, 25 ng/ml, Sigma-Aldrich, USA) and ionomycin (1 μg/ml, Sigma-Aldrich, USA) in the presence of GolgiPlug (1ul/10^6^ cells, BD Biosciences, USA) at 37°C, 5% CO2 in 24-well culture plate. After 5 h incubation, the cells were harvested and stained with PERCP-CY5.5 conjugated anti-mouse CD4 (PERCP-CY5.5-CD4, BD Biosciences). The cells were stained intracellularly with anti–IL-22, −IL-17, or –IFN-γ mAb conjugated with PE, APC, or FITC after fixation and permeabilization (BD Biosciences, eBioscience), then analyzed on a FACS-Calibur flow cytometer (BD Bioscience). FlowJo 7.6 (Treestar, USA) was used for data acquisition. Th22 cells were defined as IL-22 + IL-17-IFN-γ-CD4 +.

### Immunohistochemistry

Rabbit polyclonal antibodies against mouse IL-22R (Bioss, Beijing, China) were used as primary antibodies at a 1: 200 dilution. The heart sections were stained by using streptavidin-biotin complex kit (Boster, Wuhan, China). After the sections were rehydrated, endogenous peroxidase activity was blocked with 3% hydrogen peroxide for 10 min at room temperature. The sections were then incubated in 5% bovine serum albumin for 20 min followed by in the primary antibody at 4°C for 24 h. The sections were incubated with streptavidin-biotin complex for 20 min and visualized with 3, 3-diaminobenzidine (Boster, Wuhan, China) under a light microscope. Non-immune goat serum was used as a control. IL-22R in the cytoplasm and cytomembrane of myocardium was evaluated semi-quantitatively using Image-Pro Plus Version 6.0 (Media Cybernetics, Bethesda, MD). 5 fields from each slice were randomly selected by two pathologists to measure integrated optical density (IOD).

### Real-time RT-PCR

Total RNA of homogenized left-ventricular tissues of the mouse heart was extracted with TRIZOL Reagent® (Invitrogen, USA), and then reverse transcripted into cDNA with a Reverse Transcription kit (Takara, Dalian, China). Primers for COL1-A1, COL3-A1, MMP-9, TIMP-1 and the housekeeping gene β-actin are designed by Primer Premier 5.0 (Table 1). Real time-polymerase chain reaction (RT-PCR) was performed by using an ABI 7300 Sequence Detection System (Applied Biosystems, Foster City, CA) using SYBR green. After initial denaturation for 30 sec at 95°C, a two-step cycling procedure (95°C for 5 sec, 60°C for 31 sec) was used for 40 cycles. The relative gene expressions was normalized to the level of β-actin transcripts and quantified by the △△CT method using a 7300 System Sequence Detection software (Applied Biosystems, Foster City, CA). All reactions were performed at least in duplicate for each sample.

**Table 1:** **Sequences of primers for real-time RT-PCR**

**Molecule**	**Sequence (5’ ~ 3’)**
COL1-A1	sense: CGCCATCAAGGTCTACTGC
GenBank: 12842	anti-sense: GAATCCATCGGTCATGCTCT
COL3-A1	sense: GTGGCTCTAATGGCATCAAAG
GenBank: 12825	anti-sense: ATGTGGTCCAACTGGTCCTCTG
MMP9	sense: TGGGACCATCATAACATCAC
GenBank: 17395	anti-sense: GATACCCGTCTCCGTGCT
TIMP-1	sense: CTTGGTTCCCTGGCGTACTC
GenBank: 21857	anti-sense: ACCTGATCCGTCCACAAACAG
β-actin	sense: AATTCCATCATGAAGTGTGA
GenBank: 11461	anti-sense: ACTCCTGCTTGCTGATCCAC

### Cytokine assay

Plasma cytokine content was determined using enzyme-linked immunosorbent assays. IL-22 was measured by the Mouse IL-22 Platinum Enzyme linked immunosorbent assay (ELISA) (eBioscience, BMS6022, USA). The levels of COL1-A1, TIMP-1 and MMP9 in mice were also determined by ELISA (Cusabio Biotech, China and Boster, China). The sensitivity of ELISA kits for IL-22, COL1-A1, TIMP-1 and MMP9 was 5, 78, 39 and 5 pg/ml, respectively, and no cross-reactivity was detected. All samples were measured in triplicate.

### Statistical analysis

Data were expressed as the means ± standard deviation (SD). Statistical analyses were performed with one-way ANOVA. Correlations were determined by Spearman rank correlation coefficients. Kaplan-Meier survival curve was used to estimate the survival rate of mice and the log-rank test was used to assess the difference of the survival rate among mouse groups. All statistical analyses of data were performed using SPSS 17.0 (IBM, USA). Differences at *p* < 0.05 among the means were deemed to be statistically significant.
